# Student Doctor Network: Fake News or Facts for Emergency Medicine Applicants?

**DOI:** 10.5811/westjem.2021.10.53581

**Published:** 2022-01-05

**Authors:** Sean B. Schnarr, Victoria Gonzalez, Neeraj Chhabra

**Affiliations:** Cook County Health and Hospitals System, Department of Emergency Medicine, Chicago, Illinois

## Abstract

**Introduction:**

Residency applicants use multiple resources to guide their application process including the Student Doctor Network (SDN), a publicly available online forum for the discussion of various topics in medical education. In recent years, specialty-specific forums for residency applicants to self-report their own application information have become popular. These forums allow other applicants to review self-reported data from their peers to inform their own application process. The accuracy of this resource is unknown. To determine whether the SDN is an accurate source of information for emergency medicine (EM) applicants, we compared self-reported SDN data to objective data from the National Resident Matching Program (NRMP).

**Methods:**

We retrospectively reviewed self-reported SDN data by DO and MD candidates from EM forums for the 2014, 2016, and 2018 residency application cycles. These data were compared to the NRMP charting outcomes for each respective year.

**Results:**

A total of 360 EM applicants self-reported data on the SDN during the years reviewed. The majority of these applicants (79%) posted for the 2018 application cycle following transition to a Google Docs spreadsheet. For the first two years of analysis, mean United States Medical Licensing Examination (USMLE) scores were similar to SDN reports. For the most recent year studied, applicants who posted to SDN reported higher mean (USMLE) Step 1 (234, 95% confidence interval [CI], 233–236) and Step 2 scores (250, 95% CI, 248–251) when compared to NRMP data (231 and 241). Reported contiguous residency program ranks were similar to NRMP in all years, and the proportion indicating Alpha Omega Alpha Honor Medical Society membership was similar to NRMP only for the most recent year studied.

**Conclusion:**

Self-reporting on SDN showed a slight bias toward higher USMLE step scores in the most recent year when compared to objective NRMP data. Self-reporting on SDN has increased in recent years, but it is unknown whether this increase will lead to more accurate information for EM applicants. Given the self-reported nature of the SDN, applicants should use SDN forums with caution.

## INTRODUCTION

Medical students interested in emergency medicine (EM) have multiple resources available to assist them during their residency application process.^1^–^3^ The National Resident Matching Program (NRMP), for example, publishes data from medical students entering the match process within each medical specialty.^4^ The NRMP’s *Charting Outcomes in the Match* publications include applicants’ mean United States Medical Licensing Examination (USMLE) step scores, and Alpha Omega Alpha (AOA) Honor Medical Society status. Advisors, mentors, and other official resources provide applicants with additional information on the application process including application approaches, interview strategies, and general statistics for residency programs.^5^,^6^ Despite these resources, medical students are often unaware of how their residency application compares to their peers, leaving applicants to use other, less official, resources with undetermined accuracy.^7^

One commonly used online resource is the Student Doctor Network (SDN, www.studentdoctor.net), which offers an online forum for students, residents, and attending physicians to discuss past and current experiences with the match process, among other topics. The SDN hosts forums for its online community by subject matter spanning all stages of medical education. The forums are available for public viewing, but posting is restricted to those with an account on the website. In recent years, it has become common for residency applicants to provide self-reported data from their own residency application.^8^,^9^ These data can then be accessed by other potential applicants to evaluate the competitiveness of their own application. Applicants who use this data to inform their own application process must do so with caution, as these posts are anonymous and there is no mechanism to ensure their accuracy.

A comparison of self-reported SDN and NRMP data in the comparatively small field of radiation-oncology showed bias of aggregate self-reported test scores toward higher-scoring applicants.^9^ There are no studies to date comparing self-reported SDN data with NRMP’s published data in the larger field of EM. As SDN represents a potential source of important information for EM applicants, our goal in this study was to compare SDN data with NRMP data to determine whether self-reported SDN data is an accurate representation of the typical EM applicant.

## METHODS

This was a retrospective analysis of self-reported applicant data within EM forums on the SDN. Those who reported on the SDN either used the forum system on annual threads for EM applicants or, in the case of the 2017–2018 application cycle, a Google Docs spreadsheet (Google LLC, Mountain View, CA) was created that allowed users to anonymously add their own data without creating an account on the SDN. Links to this spreadsheet were posted to the SDN and the website Reddit (www.reddit.com) (Reddit Inc., San Francisco, CA).^10^ With the forum system, respondents replied to the original thread with a post to provide their application information in a structured format under their SDN username. These were subsequently aggregated by the researchers. The spreadsheet allowed anonymous users to provide the same structured data in an already aggregated format.

We performed data collection and analysis for the 2014, 2016, and 2018 application cycles because those were the years with corresponding NRMP publications.^11^–^15^ For the purposes of this study, DO and MD applicants were pooled. Given the different application experiences of international medical graduates (IMG) applying for EM residency, such as the average number of applications submitted, we excluded IMGs from analysis.^16^,^17^

The variables collected from the SDN included those available in NRMP publications such as USMLE Step 1 and 2 scores, AOA status, and number of contiguous ranks as well as those commonly included in SDN forums, including number of residency applications and number of accepted interview invitations. Given the self-reported nature of the SDN, there were missing data points that were not included in analysis. We obtained comparison data from NRMP *Charting Outcomes in the Match* for 2014, 2016, and 2018. The NRMP provides means and proportions but not distributions, so we did not perform direct statistical comparisons with SDN data. We analyzed data by descriptive statistics using Microsoft Excel (Microsoft Corporation, Redmond, WA). Descriptive data are reported as means with 95% confidence intervals (CI) to match NRMP reports, where applicable, while medians and interquartile ranges (IQR) were used for non-parametric data not reported by NRMP. This study was reviewed by our institution’s institutional review board and deemed exempt because it used de-identified and publicly available information.

**Population Health Research Capsule**

What do we already know about this issue?

*Medical students often use various online resources to help guide their residency application process*.

What was the research question?

*How accurate is self-reported data regarding emergency medicine applicants on the Student Doctor Network (SDN)?*

What was the major finding of the study?

*Self-reported data on SDN showed slight bias toward higher USMLE scores compared to objective NRMP data*.

How does this improve population health?

*Online resources are often relied on heavily by medical students, but should be used with caution when applying to emergency medicine residency*.

## RESULTS

In total, there were 360 applicants with self-reported information on the SDN in the years 2014, 2016, and 2018, representing 7.3% of all EM applicants during the time period. The majority (79%) of SDN applicants self-reported in the 2018 application cycle, which used a Google Docs spreadsheet instead of a typical SDN forum. This sample represented 14.5% of all EM applicants for that year. The mean USMLE Step 1 and Step 2 scores reported by applicants was 235 and 249, respectively. Table 1 shows cumulative, self-reported SDN applicant data for the included years. Table 2 shows a comparison of SDN data by year with corresponding data reported by the NRMP. As the NRMP data represents true population totals, 95% CIs were not calculated. In general, those who posted on the SDN had similar USMLE step scores and a similar number of contiguous ranks. For 2018, however, aggregated USMLE step scores from the SDN showed a higher average than reported by the NRMP. The mean number of applications submitted per applicant was 53 with a median of 45, indicating a positive skew.

## DISCUSSION

From the information provided by EM applicants on the SDN and those compiled by the NRMP, the mean USMLE Step 1 and Step 2 scores reported for applicants by the NRMP was similar to those self-reported by applicants. While the average USMLE Step 1 and Step 2 scores were higher on the SDN self-reported data than the NRMP for all years compared, they were typically within the 95% CI of the mean. Exceptions to this were noted in 2018, indicating that for this year, the SDN had a bias toward higher scoring applicants. One possible explanation for this discrepancy is that applicants with lower scores may be less willing to publicly disclose their test scores, even anonymously.

Applicants should interpret anonymously self-reported examination scores with caution. The average number of contiguous ranks between the SDN and NRMP, however, were similar in all years studied. According to the NRMP, applicants with 12 contiguous ranks had approximately a 95% probability of matching, which is a valuable data point for future applicants.^4^ Given that most categories, in aggregate, appear similar to NRMP data while some show important differences, it is unclear how applicants should best use the SDN as a potential data source to inform their own application process.

With the use of Google Docs in 2018, there was a nine-fold increase in the number of users posting data using the SDN compared to 2016. This has been observed in previous studies comparing these two sources and is likely due to the ease of use, anonymity, and ability to access the spreadsheet from either the SDN or Reddit.^8^,^9^ As more users contribute in future years, it is possible that the differences noted between SDN and NRMP data will decrease, as was seen with the percentage of applicants that claimed AOA status. Alternatively, given the ease with which users can anonymously post, some posts on the Google Doc may not be accurate and the spreadsheet may be unavailable periodically due to inappropriate and/or offensive posts and necessary maintenance.

There was considerable variability in the reported number of residency applications submitted by SDN users, with an interquartile range of 35 to 62.25 applications. It is unknown whether the range among applicants was due to counsel from advisors, perceived strength or weakness of individual applications, or a combination of the two. This is an important consideration for applicants as medical students are applying to more residency programs, often at significant personal cost.^18^,^19^ At the current 2021 Electronic Residency Application Service (ERAS) fee structure, the average number of applications from the current study (53) would cost $1187 per applicant.^20^ For medical students, most with limited to no income, this cost is unreasonable but may be deemed necessary to “keep up” with their peers. While advisors may counsel against an inordinate number of applications per applicant, students may be influenced by noting how many programs their peers report on the SDN that they are applying to. Applicants, advisors, and ERAS should explore ways to address the increasing number of applications and limit the costs of the application process to avoid placing applicants from less privileged financial backgrounds at a competitive disadvantage.

## LIMITATIONS

There were several limitations to this study. For the 2018 application cycle, users did not self-report data on successful matching; so this was excluded from analysis. Although the anonymous forum dramatically increased the number of users who posted information, it is likely that many users simply stopped using the website after a successful match. As both NRMP aggregated data and SDN data are anonymous, direct comparisons of these data in individual applicants was not possible. Similarly, data collection techniques significantly differ between the two sources. Further, given the small sample size from the SDN, conclusions regarding its accuracy should be tempered.

## CONCLUSION

Self-reported EM applicant data on the Student Doctor Network is similar to data provided by the NRMP with a bias in recent years toward higher self-reported standardized test scores. With the emergence of Google Docs as a centralized and more anonymous avenue for self-reporting data, a dramatic increase in applicants providing information was noted for the most recent application cycle. Whether this trend will provide more accurate data for potential EM residency applicants remains to be seen.

## Figures and Tables

**Table 1:** Summary of Student Doctor Network data from 2014, 2016, and 2018.

Variable	Value
Mean number of contiguous ranks (95% CI)	12 (11–13)
Mean USMLE Step 1 score (95% CI)	235 (233–237)
Mean USMLE Step 2 score (95% CI)	249 (248–251)
AOA, n (%)	
Yes	29 (8%)
No/Unknown	331 (92%)
Couples match, n (%)	
Yes	6 (2%)
No	0 (0%)
Unknown	354 (98%)
Any research, n (%)	
Yes	24 (7%)
No	47 (13%)
Unknown	289 (80%)
Median number of applications submitted (IQR)	45 (35–62.25)
Median number of interviews received (IQR)	20 (12.25–26.5)
Median number of interviews attended (IQR)	13 (6–16.75)
Matched on rank list, n (%)	
1	12 (3%)
2	0
3	6 (2%)
4	1(0.3%)
5	0
6	1(0.3%)
7	1(0.3%)
Unknown	339 (94%)

*CI*, confidence interval; *USMLE*, United States Medical Licensing Examination; *AOA*, Alpha Omega Alpha Honor Medical Society; *IQR*, interquartile range.

**Table 2:** Comparison of Student Doctor Network and National Resident Matching Program data.

	SDN 2014	NRMP 2014	SDN 2016	NRMP 2016	SDN 2018	NRMP 2018
Applicants (n)	42	1,371	31	1,576	286	1,972
USMLE Step 1 score (Mean, 95% CI)	235 (229–240)	230	238 (234–243)	233	234 (233–236)	231
USMLE Step 2 score (Mean, 95% CI)	245 (241–250)	243	248 (242–254)	245	250 (248–251)	241
Number of contiguous ranks (Mean, 95% CI)	12 (11–13)	11.9	12 (10–13)	11.2	12 (10–14)	11
AOA Membership* (%)	27%	12%	33%	13%	13%	12%

* Applicants not indicating an AOA status were presumed to not be AOA members.

*SDN*, Student Doctor Network; *NRMP*, National Resident Matching Program; *USMLE*, United States Medical Licensing Examination; *CI*, confidence interval; *AOA*, Alpha Omega Alpha Honor Medical Society.
